# The Impact of Implementing an Evidence‐Based Intrapartum Care Quality Improvement Program on Childbirth Outcomes for Laboring Women: An Interrupted Time‐Series Analysis

**DOI:** 10.1155/jonm/2066972

**Published:** 2026-08-14

**Authors:** Wenli Zhu, Hui Min, Yaming Dai, Chunxiang Zhu, Xiaojiao Wang, Chunyi Gu

**Affiliations:** ^1^ Department of Nursing, Obstetrics and Gynecology Hospital of Fudan University, Shanghai Key Lab of Reproduction and Development, Shanghai Key Lab of Female Reproductive Endocrine Related Diseases, Shanghai, China, fudan.edu.cn; ^2^ Department of Nursing, Xinhua Hospital Affiliated to Shanghai Jiaotong University School of Medicine, Shanghai, China, xinhuamed.com.cn; ^3^ Department of Obstetrics, Obstetrics and Gynecology Hospital of Fudan University, Shanghai, China, fudan.edu.cn; ^4^ School of Public Health, NHC Key Laboratory of Health Technology Assessment, Fudan University, Shanghai, China, fudan.edu.cn

**Keywords:** cesarean section, childbirth experience, intrapartum care, labor, midwife

## Abstract

**Objectives:**

To evaluate the impact of implementing an evidence‐based intrapartum care quality improvement program on childbirth outcomes for laboring women.

**Methods:**

A quasiexperimental interrupted time series (ITS) study using routinely collected hospital data was conducted at the Obstetrics and Gynecology Hospital of Fudan University, Shanghai, China. The study period spanned from November 1, 2021, to December 31, 2022, with a 4‐month preparation phase (April 1, 2022, to July 31, 2022) excluded from analysis. A total of 422 women were included (211 preintervention and 211 postintervention), aggregated into 20 biweekly time points (10 preintervention and 10 postintervention). Key measures included intrapartum cesarean section (ICS) rates, oxytocin use, duration of labor, and childbirth experience scores. Sociodemographic and clinical data were extracted from routine records, and childbirth experience was assessed using the Childbirth Experience Questionnaire (CEQ 2.0).

**Results:**

Prior to the intervention, the ICS rate increased by 2.057% per biweekly time point (*p* = 0.015). After implementation, the ICS rate showed a decreasing trend, with a slope reduction of 2.782% compared with the preintervention trend (*p* = 0.018), resulting in an overall postintervention slope of −0.725% per biweekly time point. The estimated contribution of the intervention to ICS reduction was −22.69%. Compared with the preintervention group, women in the intervention phase had lower oxytocin use (71.09% vs. 80.09%, *p* < 0.05), shorter total labor duration (median 456.5 min [265.0–602.5] vs. 472.5 min [280.0, 670.0], *p* < 0.05), and higher childbirth experience scores (72.23 ± 9.76 vs. 69.27 ± 8.84, *p* < 0.05).

**Conclusion and Implications for Nursing Management:**

This quality improvement program effectively optimized intrapartum care through reduced medical interventions and enhanced childbirth experiences. Successful scaling up requires institutional commitment to midwifery continuity models, context‐adaptive implementation strategies, and sustained investment in midwifery training and outcome monitoring systems to ensure equitable and woman‐centered care delivery.

## 1. Background

Childbirth represents a transformative and pivotal event in a woman’s life, characterized by the complex interplay of physiological, psychological, and social factors. As the cornerstone of safe midwifery practice, intrapartum care—defined as the care given by healthcare providers for childbearing women during labor and delivery [1]—critically influences both maternal and neonatal outcomes. Global healthcare systems continue to prioritize quality improvement initiatives in intrapartum care, recognizing that effective monitoring of labor and childbirth, along with the early identification and treatment of complications, are essential for preventing adverse perinatal outcomes [2]. The 2018 WHO recommendations on intrapartum care marked a paradigm shift by emphasizing evidence‐based practices that optimize clinical outcomes and enhance women’s childbirth experiences [3]. These recommendations advocate for scientific approaches that improve care efficacy while respecting maternal autonomy, a dual focus that has gained increasing recognition in contemporary clinical practice.

The transition to evidence‐based intrapartum care models reflects a fundamental transformation in healthcare delivery, prioritizing interventions validated through rigorous scientific inquiry over traditional practice patterns. Such models integrate the best available research with clinical expertise to standardize care processes, reduce unwarranted variations in practice, and improve patient satisfaction [3]. However, evidence suggests that the implementation of evidence‐based practice remains inconsistent in clinical settings, with healthcare professionals facing barriers related to knowledge, attitudes, and organizational support [4]. Despite technological advancements and an expanding body of medical knowledge, significant disparities persist in the quality of intrapartum care across healthcare settings. These variations arise from multifactorial influences, including institutional protocols, clinician preferences, and regional disparities in resource allocation [5]. This heterogeneity underscores the imperative for standardized, evidence‐driven intrapartum care frameworks that ensure consistent quality across diverse clinical contexts [6]. In addition to clinical interventions, growing evidence highlights the importance of psychosocial determinants in shaping childbirth experiences. Factors such as social support, emotional wellbeing, and perceived control during labor are strongly associated with maternal satisfaction and overall quality of life during pregnancy [7]. Continuous labor support, respectful communication, and reduced unnecessary interventions have been identified as key pathways linking intrapartum care practices with improved childbirth experiences and maternal wellbeing [3]. These findings underscore the need for intrapartum care models that integrate both clinical effectiveness and psychosocial support.

Globally, various intrapartum care interventions have demonstrated meaningful improvements in maternal outcomes. For instance, the WHO intrapartum care guideline focuses on maternal and neonatal health outcomes, intervention frequencies, and patient satisfaction. The implementation of this guideline has yielded measurable improvements, including reduced cesarean section rates, increased spontaneous vaginal deliveries, and enhanced maternal satisfaction and sense of safety [8]. Similarly, a 2024 Cochrane systematic review demonstrated that midwife continuity of care models result in fewer medical interventions, reduced cesarean rates, and higher maternal satisfaction compared with other models [9]. The NHS Birthplace Study in the United Kingdom revealed that midwife‐led care models resulted in fewer medical interventions and higher satisfaction for low‐risk pregnancies [10]. In the United States, intrapartum care improvement projects have successfully reduced cesarean rates and increased patient satisfaction through targeted interventions [11]. Emerging evidence suggests that innovative approaches, such as patient‐centered care, continuous midwifery support, and psychological support [12], are key components for enhancing the overall childbirth experience [3]. However, the generalizability of these findings remains constrained by contextual factors including healthcare infrastructure and cultural perceptions of childbirth.

In recent years, China’s evolving maternity care landscape presents unique implementation challenges despite recent improvements in service coverage. Routine practice in many Chinese maternity settings has been characterized by variability in labor monitoring, high reliance on oxytocin augmentation, limited continuous labor support, and inconsistent decision thresholds for intrapartum cesarean section (ICS). These challenges were compounded by fragmented continuity of care, heterogeneous midwifery practices, and limited implementation of standardized evidence‐based protocols [13]. This context underscores the urgent need for context‐adapted, evidence‐based intrapartum care interventions that reconcile global best practices with local implementation realities. Therefore, context‐adapted quality improvement strategies are needed to standardize labor management, optimize intervention use, and strengthen supportive care.

To address these gaps, this study implemented an evidence‐based intrapartum care quality improvement program and evaluated its effectiveness using a quasiexperimental interrupted time series (ITS) design. The program targeted key aspects of labor management, including labor monitoring, oxytocin use, decision‐making for ICS, and supportive care during childbirth. We examined changes in ICS rates, oxytocin use, duration of labor, and childbirth experience before and after implementation. By analyzing outcome trajectories over time, this study aimed to assess the effectiveness of the program in improving intrapartum care quality and maternal childbirth experience within the Chinese maternity care context.

## 2. Methods

### 2.1. Study Design

A quasiexperimental ITS design was used to evaluate the impact of an evidence‐based intrapartum care quality improvement program on childbirth outcomes [14]. The ITS framework enabled a longitudinal assessment of outcomes before and after the implementation of the program, utilizing routinely collected hospital data from November 1, 2021, to December 31, 2022, at the Obstetrics and Gynecology Hospital of Fudan University, Shanghai, China. The study period was divided into biweekly intervals, generating a total of 20 time points: 10 preintervention points (November 1, 2021–March 31, 2022) and 10 postintervention points (August 1–December 31, 2022). The preparation phase, spanning April 1 to July 31, 2022, was excluded from the segmented regression analysis. Aggregated outcome measures were calculated for each time point to assess level and slope changes following the intervention. Ethical approval was obtained from the Ethics Committee of the Obstetrics and Gynecology Hospital, Fudan University (OGHEC2021107). All potential participants were given written information about the study’s purpose, their rights to voluntary participation, and the option to withdraw at any time. Written informed consent was obtained prior to enrollment.

### 2.2. Study Population

Women with low‐risk pregnancies who were admitted for delivery at the study hospital were eligible for inclusion. The inclusion criteria were as follows: (1) women aged between 18 and 49 years; (2) healthy women at term (37–41.6 gestational weeks); (3) singleton, vertex presentation; and (4) with spontaneous onset of labor. The exclusion criteria included (1) a history of mental illness; (2) visual disturbances or hearing disabilities; and (3) medical and/or surgical complications such as heart disease, hypertension, and gestational diabetes mellitus requiring control by medication.

Within each biweekly interval, eligible women were consecutively recruited from admissions to the labor and delivery unit. Individual‐level data were collected for all eligible women during the study period.

### 2.3. Time Segmentation and Data Collection

The study period (November 1, 2021, to December 31, 2022) was divided into three phases: preintervention, preparation, and intervention. The primary ITS unit of analysis was the biweekly aggregated outcome. For each time point, ICS rate, oxytocin use rate, and other outcomes were calculated as the proportion of eligible women within that interval. Individual‐level data were used to compute outcome measures for each two‐week time point, including the ICS rate. Each participant contributed to a single time point based on their admission date, and all eligible women within each interval were included. The number of women per biweekly time point ranged from 15 to 28 in the preintervention phase and from 16 to 25 in the postintervention phase, with an average of 21.1 women per time point in each phase.

#### 2.3.1. Preintervention Phase

Baseline data were collected from November 1, 2021, to March 31, 2022, representing routine intrapartum care prior to implementation of the quality improvement program.

#### 2.3.2. Preparation Phase

From April 1, 2022, to July 31, 2022, an implementation preparation phase was conducted. Training materials were developed, and healthcare providers received structured training on the evidence‐based intrapartum care quality improvement program, including labor monitoring, oxytocin use, decision thresholds for ICS, and supportive care practices. Data from this phase were not included in segmented regression analysis but were retained for descriptive purposes.

#### 2.3.3. Intervention Phase

The intervention was implemented from August 1, 2022, to December 31, 2022. Data were continuously collected during this period.

### 2.4. Data Collection

Maternal sociodemographic and clinical data were collected from admission to discharge. Baseline characteristics included age, education level, family income, parity, and gravida, obtained using structured questionnaires. Clinical outcomes included the duration of labor, rupture of membranes, oxytocin use, labor analgesia, mode of delivery, neonatal birth weight, postpartum hemorrhage, and abnormal labor. These data were recorded using standardized data collection forms based on routinely documented clinical records.

Women’s childbirth experience was assessed using the Childbirth Experience Questionnaire (CEQ 2.0). The questionnaire was administered electronically via Questionnaire Star within 24–48 h prior to hospital discharge by trained research staff. All enrolled women (*N* = 422) completed the CEQ 2.0 with no missing responses; therefore, no imputation or exclusion for missing CEQ data was performed. Clinical variables were extracted from routine medical records. The overall missing rate for key clinical variables was < 5%, and complete‐case analysis was performed.

### 2.5. Implementation

#### 2.5.1. Control Group

Women in the control group received conventional intrapartum care from November 1, 2021, to March 31, 2022. Childbirth care and labor monitoring were provided by shift midwives and obstetricians throughout the first, second, and third stages of labor, as well as the first 2 h postpartum. No midwife was exclusively assigned to a single woman, and one midwife typically cared for multiple laboring women simultaneously. Women received care in shared labor and delivery rooms without family companionship. During the second stage of labor, the shift midwife provided routine instruction for pushing. Immediate umbilical cord clamping was routinely performed after birth, followed by transfer of the newborn to the neonatal ward for approximately 2 h of observation. Afterward, the newborn was returned to the mother. During the first 2 h postpartum, routine health education focusing on perineal care was provided.

#### 2.5.2. Intervention Group

Women in the intervention group received care through an evidence‐based intrapartum care quality improvement program, which introduced a midwife‐led continuity‐of‐care model with individualized support in a single labor and delivery room.

Program development: The program was developed based on nine clinical guidelines [3, 15–22]. A panel of 22 experts in obstetrics, midwifery, maternal–child health, and evidence‐based practice reviewed the draft. The expert familiarity coefficient was 0.927, judgment basis 0.991, and authority coefficient 0.959. The finalized program consisted of six components: intrapartum care principles, first‐stage care, second‐stage care, third‐stage care, neonatal care, and early postpartum care. Subsequently, a quality improvement team was established, including the Director of Nursing, Chief Obstetric Nurse Manager, Director of the Labor Unit, and six senior midwives. Using the JBI PIPOST framework, barriers to implementation were identified, including inconsistent labor monitoring practices, variable oxytocin use, limited labor companionship, and lack of standardized supportive care.

### 2.6. Intervention Components

The evidence‐based intrapartum care program introduced the following practices.

Newly introduced practices:

• One‐to‐one midwife‐led continuous labor support
• Family companionship during labor
• Freedom of movement and upright labor positioning
• Structured nonpharmacological pain relief (birth ball, breathing, and massage)
• Standardized delayed umbilical cord clamping (≥ 60 s)
• Immediate skin‐to‐skin contact after birth
• Early breastfeeding initiation guidance

Previously available but inconsistently implemented:

• Continuous labor monitoring using standardized criteria
• Restrictive oxytocin augmentation with clinical indications
• Individualized pushing guidance
• Respectful communication and shared decision‐making
• Postpartum education covering recovery and breastfeeding

Preimplementation preparation: Prior to implementation, educational materials including protocols, videos, and manuals were distributed to all midwives in the labor and delivery unit. Structured training sessions were conducted covering labor monitoring, supportive care, oxytocin use, cesarean decision thresholds, and neonatal care practices. Training included lectures, simulations, and case discussions. Training completion was required for all participating midwives. Competency assessments were conducted following training, and only midwives who passed the assessment were allowed to deliver intervention care.

### 2.7. Implementation Fidelity

To ensure implementation fidelity, multiple monitoring strategies were applied, which included tracking midwives training completion, conducting weekly adherence audits with standardized checklists, and performing random observational assessments by senior staff. These efforts were bolstered by monthly feedback meetings and a comprehensive documentation review of labor support and intervention practices. Key fidelity indicators focused on the proportion of women receiving one‐to‐one midwife support, the utilization of upright labor positions, delayed cord clamping, immediate skin‐to‐skin contact, and the restricted use of oxytocin. Implementation was conducted across all shifts. To minimize variability, trained midwives were scheduled to ensure coverage during day and night shifts. When staffing shortages occurred, temporary interruption of 1:1 care was documented. Overall, one‐to‐one midwife support was maintained for the majority of labor duration, with brief interruptions primarily during emergency situations or shift handovers.

### 2.8. Minimizing Spillover Effects

To reduce contamination between control and intervention periods, implementation was introduced only after completion of baseline data collection. Training and dissemination of intervention materials were restricted to the preparation phase. During the preintervention phase, staff followed routine care protocols without exposure to the structured program. Additionally, audit checklists and intervention documentation were activated only after the formal implementation date (August 1, 2022).

Implementation process: In this evidence‐based intrapartum care quality improvement program (Table 1), a designated midwife was tasked with providing continuity of care and support for the participating woman from the onset of labor. The woman’s family members were actively involved throughout the entire labor companionship process. Women were allowed and encouraged to move freely during labor, with guidance on the advantages of adopting upright positions to facilitate labor progression. The midwife instructed the woman to select comfortable positions while discouraging prolonged supine positions. Nonpharmacological pain relief measures including birth balls, doula care, breathing techniques, and lower back massages were offered, alongside informed discussion regarding pharmacological pain relief options. Following complete cervical dilation, the midwife assessed the woman’s readiness and preference for pushing, the fetal descent, and variations in the fetal heart rate. Delayed umbilical cord clamping for at least 60 s was performed for full‐term or premature infants exhibiting vitality at birth (high quality, strongly recommended). The newborn baby was promptly placed on the mother’s chest or abdomen. Early postpartum skin‐to‐skin contact and guidance for breastfeeding initiation were provided for the newborn in good condition. During the first 2 h postpartum, the designated midwife provided a comprehensive series of health education to the mother, covering topics such as perineal care, early postpartum activities, nutritional support, prevention of postpartum infections, and postpartum contraception and birth spacing.

**TABLE 1:** Information details between the intervention group and the control group.

	Key elements	Control group	Intervention group
The entire childbirth process	Companion support	The woman underwent labor progress, delivery, postpartum observation, and care in a shared labor and delivery room by the shift midwife and obstetrician, without family members present during childbirth.	The designated midwife and the woman’s family members participated in providing companionship, childbirth care and support in a single labor and delivery room.
	Emotional support	Routine verbal greeting during labor, but without using specific tools to assess the woman’s emotional and psychological status and needs.	The woman’s emotional and psychological status and needs were assessed upon admission to the labor and delivery unit and 2 h postpartum using a simple maternal mental health screening tool for childbirth.
	Continuous care	No fixed midwives cared for the laboring woman during the process of labor and birth.	One‐to‐one continuous care was provided by one designated midwife in the family‐centered labor and delivery room.
	Referral for abnormal labor	Lack of clear referral criteria for abnormalities occurring in either the woman or the fetus/newborn.	A clear referral pathway was established if any abnormalities of labor happened to either the woman or the fetus/newborn.

The first stage of labor	Pain relief measures	Routine pain assessments using the Visual Analogue Scale (VAS) were performed for the laboring woman.	Based on the VAS assessments, multiple pain relief measures were offered to the laboring woman, including provision of related health education and support, performing various non‐pharmacological pain relief measures such as birthing balls, doula care, breathing techniques, and lower back massages, alongside information on pharmacological pain relief options.
	Maternal positioning and activity during labor	The laboring woman typically remained in a resting position in bed, with inadequate guidance provided to engage ambulation or position changes.	The designated midwife was responsible for encouraging the woman to engage in free movement during labor.

The second stage of labor	Pushing technique	When the woman’s cervix was fully dilated, the shift midwife instructed the woman to perform Valsalva pushing during contractions by holding her breath and bearing down.	When the woman’s cervix was fully dilated, the designated midwife encouraged the woman to choose spontaneous pushing or delayed pushing based on her preference. The midwife assessed the presence of maternal bearing‐down urge, fetal descent, and fetal heart rate patterns. If an active bearing‐down urge was present, the midwife guided the woman to begin pushing. In cases where no pushing was felt and the fetal station remained above “+3” level without signs of fetal distress, delayed pushing was recommended. During this period, the woman was allowed to rest or change positions. Pushing was initiated when the fetal presenting part reached “+3” station, and the woman developed a strong, involuntary bearing‐down reflex.

The third stage of labor	Early essential newborn care	Immediate cord clamping was performed following delivery. The newborn was then transferred to the neonatal unit for standard 2 h observation.	Delayed umbilical cord clamping was performed for at least 60 s for newborns who were in good conditions and without complications following birth. Immediate skin‐to‐skin contact was initiated postpartum, while the attending midwife provided guidance on early breastfeeding initiation.

2 h postpartum	Postnatal health education	Within 2 h after childbirth, the midwife provided routine oral health education to the woman mainly on postnatal perineal care.	Within 2 h after childbirth, the designated midwife provided comprehensive health education covering perineal care, early ambulation, nutritional guidance, postpartum infection prevention, and postpartum contraceptive counseling. The education was delivered through verbal instructions, educational videos and informational brochures.

### 2.9. Outcome Measures

This study evaluated primary and secondary outcomes to assess the impact of an evidence‐based intrapartum care quality improvement program. The ICS rate served as the primary indicator of intervention effectiveness. A reduction in the ICS rate indicated a positive impact of the intervention. Secondary outcomes included intervention contribution ratio (*C*), childbirth outcomes, and women’s childbirth experience. The measure of the intervention *C* quantified the contribution of the intervention to the reduction in the ICS rate. The formula for *C* is as follows: *C* = [*X*_*j*_ − [*X*_*i*_ + (*Y*_*j*_ − *Y*_*i*_)]]/[*X*_*i*_ + (*Y*_*j*_ − *Y*_*i*_)] × 100%. *Y*_*i*_ indicates the ICS rate at the start of the preintervention period; *Y*_*j*_ indicates the ICS rate at the end of the preintervention period; *X*_*i*_ indicates the ICS rate at the start of the intervention period; and *X*_*j*_ indicates the ICS rate at the end of the intervention period. A negative value of C indicates that the observed ICS rate at the end of the intervention period is lower than the counterfactual rate, implying a beneficial reduction attributable to the intervention. Clinical data were extracted from routinely collected hospital records by the research team, who were not involved in providing intrapartum care. To measure women’s childbirth experience, we adopted the CEQ 2.0 translated by Peipei Liao [23]. This questionnaire encompasses four dimensions: own capacity, professional support, perceived safety, and participation. It consists of 25 items, with the first 22 items using a 4‐point rating scale, ranging from “*strongly disagree*” (1 point) to “*strongly agree*” (4 points), with negatively worded items reverse‐scored. The last three items utilize a visual analog scale: 0–4 scores (1 point), 5‐6 scores (2 points), 7‐8 scores (3 points), and 9‐10 scores (4 points). The total scores range from 25 to 100 points, with higher values indicating more positive childbirth experiences.

### 2.10. Statistical Analysis

Descriptive data were summarized using frequency and percentage. To compare differences between two groups, *t*‐tests and chi‐square tests were applied. The ITS analysis was modeled using a linear regression model that incorporated three time‐related variables. The resulting regression coefficients estimated both the level change and the slope change before and after the intervention [24]. The model is defined as follows: *Y* *t* = *β*_0_ + *β*_1_ × time + *β*_2_ × intervention + *β*_3_ × time‐after‐intervention + *ε*_*t*_. In this equation, *Y* *t* represents the outcome variable, specifically the ICS rate observed in the study. *β*_0_ denotes the estimated level of the ICS rate prior to the implementation of the evidence‐based intrapartum care quality improvement program. *β*_1_ reflects the estimated trend in the ICS rate before the intervention, representing the slope of the rate during this period. *β*_2_ represents the immediate level change in the outcome following the intervention. *β*_3_ signifies the estimated change in the trend of the ICS rate postintervention, illustrating how the slope of the rate evolves after the intervention. *β*_1_ + *β*_3_ represents the overall trend (slope) of the ICS rate after the intervention’s implementation. Finally, *ε*_*t*_ denotes the residual at time t, which accounts for the variation in the outcome variable that is not explained by the model. This equation effectively illustrates the changes in the ICS rate throughout the study period, encompassing the preintervention trend, the direct impact of the intervention, and postintervention trend changes.

## 3. Results

### 3.1. Characteristics of the Participants

Between November 1, 2021, and December 31, 2022, a total of 422 laboring women were enrolled in the study. We recruited 211 participants in the preintervention phase (baseline period) and 211 in the intervention phase. Baseline characteristics were comparable between groups, with the exception of parity which showed a statistically significant difference (*p* < 0.05). All other demographic and clinical variables demonstrated no significant intergroup differences (Table 2).

**TABLE 2:** Basic characteristics of participants.

Characteristics	Total (*N* = 422)	Control group (*n* = 211)	Intervention group (*n* = 211)	Statistic	*p* value
Maternal age ($\overset{¯}{x}\pm s$, years)	31.46 ± 3.54	31.69 ± 3.65	31.22 ± 3.39	1.385^∗∗^	0.168
Education level				0.800^∗^	0.670
Secondary school	13 (3.08)	5 (2.37)	8 (3.79)		
Undergraduate	371 (87.91)	186 (88.15)	185 (87.68)		
Postgraduate	38 (9.01)	20 (9.48)	18 (8.53)		
Occupation type				0.398^∗^	0.528
Light physical labor	377 (89.34)	186 (88.15)	191 (90.52)		
Moderate physical labor	45 (10.66)	25 (11.85)	20 (9.48)		
Household monthly income (CNY yuan)				0.555^∗^	0.907
< 5000	5 (1.18)	3 (1.42)	2 (0.95)		
5000–7999	174 (41.23)	89 (42.18)	85 (40.28)		
8000–10,000	206 (48.82)	102 (48.34)	104 (49.29)		
> 10,000	37 (8.77)	17 (8.06)	20 (9.48)		
Parity				3.973^∗^	0.046
Primipara	374 (88.63)	180 (85.31)	194 (91.94)		
Multipara	48 (11.37)	31 (14.69)	17 (8.06)		
Gestational weeks ($\overset{¯}{x}\pm s$, weeks)	39.30 ± 1.17	39.25 ± 1.21	39.35 ± 1.13	−0.877^∗∗^	0.381

^∗^Denotes chi‐square test.

^∗∗^Represents *t* test.

### 3.2. Childbirth Outcome Measures

Women participating in the evidence‐based intrapartum care quality improvement program were less likely to receive oxytocin infusion compared with those in the routine care group (71.09% versus 80.09%, *p* < 0.05). The total duration of labor was shorter in the intervention group (median 456.5 min [265.0, 602.5]) than that in the control group (median 472.5 min [280.0, 670.0]) (*p* < 0.05). Additionally, the third stage of labor was also significantly shorter in the intervention group (*p* < 0.001). No significant differences were observed between the two groups in the other childbirth outcome variables (all *p* > 0.05) (Table 3).

**TABLE 3:** Childbirth outcomes between the two groups.

Variables	Control group (*n* = 211)	Intervention group (*n* = 211)	Statistic	*p* value
Membrane rupture			0.038^∗^	0.846
Artificial rupture of membrane	104 (49.29)	101 (47.87)		
Spontaneous rupture of membrane	107 (50.71)	110 (52.13)		
Use of oxytocin			4.161^∗^	0.041
Yes	169 (80.09)	150 (71.09)		
No	42 (19.91)	61 (28.91)		
Duration of labor [min,M(P_25_, P_75_)]				
Total duration of labor	472.5 (280.0, 670.0)	456.5 (265.0, 602.5)	2.070^∗∗^	0.038
First stage of labor	428.0 (250.0, 603.5)	412.5 (220.0, 598.0)	‐0.286^∗∗^	0.775
Second stage of labor	36.5 (25.0, 60.0)	31.0 (20.0, 55.0)	1.644^∗∗^	0.100
Third stage of labor	7.5 (4.5, 9.5)	5.0 (4.0, 8.0)	6.578^∗∗^	< 0.001
Perineal condition^∗∗∗^			1.449^∗^	0.694
Unruptured	14 (8.92)	21 (12.21)		
First‐degree tear	112 (71.34)	119 (69.19)		
Second‐degree tear	3 (1.91)	5 (2.91)		
Episiotomy	28 (17.83)	27 (15.69)		
Labor analgesia			1.657^∗^	0.198
Yes	194 (91.94)	185 (87.68)		
No	17 (8.06)	26 (12.32)		
Neonatal birth weight			0.212^∗^	0.899
< 2500 g	5 (2.37)	4 (1.90)		
[2500, 4000) g	200 (94.79)	202 (95.73)		
≥ 4000 g	6 (2.84)	5 (2.37)		
Postpartum hemorrhage			0.454^∗^	0.500
Yes	6 (2.84)	3 (1.42)		
No	205 (97.16)	208 (98.58)		
Abnormal neonatal referral			0.540^∗^	0.463
Yes	45 (21.33)	38 (18.01)		
No	166 (78.67)	173 (81.99)		
Abnormal labor			0.342^∗^	0.559
Yes	106 (50.24)	99 (46.92)		
No	105 (49.76)	112 (53.08)		

^∗^Denotes Chi‐square test.

^∗∗^Represents *Z* test.

^∗∗∗^The variable was assessed only among women who experienced vaginal delivery.

### 3.3. Interrupted Time Series of ICS

**FIGURE 1:**
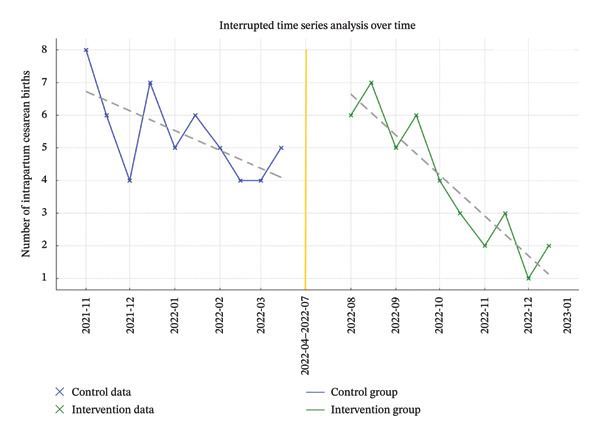
Interrupted time‐series analysis of the number of intrapartum cesarean sections.

**FIGURE 2:**
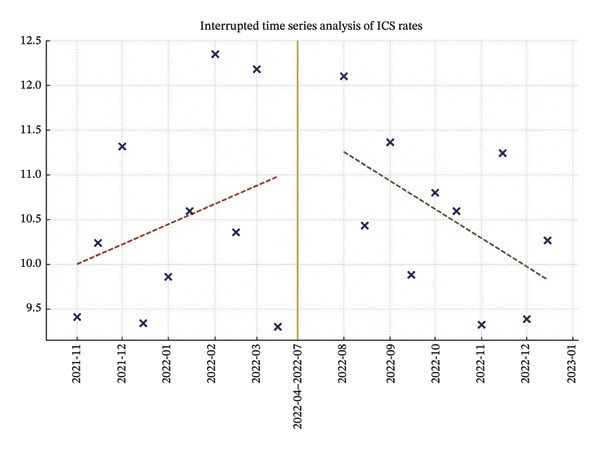
Interrupted time‐series analysis of the rate of intrapartum cesarean sections.

Prior to and following the implementation of the evidence‐based intrapartum care quality improvement program, a time‐series analysis was conducted on the indicator of the ICS rate. To ensure the accuracy of the ITS analysis, the Cumby–Huizinga test was applied to assess first‐order auto correlation. The results indicated no auto correlation in the regression model concerning the number and rate of ICS deliveries (*p* > 0.05). The period from April 1, 2022, to July 31, 2022, was defined as the implementation preparation phase. These time points were excluded from the segmented regression model to avoid contamination during program rollout. Segmented regression analyses were, therefore, conducted using preintervention (November 1, 2021–March 31, 2022) and postintervention (August 1, 2022–December 31, 2022) time points. The regression model outputs are presented in Table 4. Following implementation of the quality improvement program, the trend of ICS number and rate showed a significant reduction (*p* < 0.05) (Figures 1 and 2). Prior to the intervention, the ICS rate demonstrated an increasing trend, rising by an average of 2.057% every 2 weeks (*p* = 0.015). After implementation, the ICS rate showed a decreasing trend, with a slope reduction of 2.782% compared with the preintervention trend (*p* = 0.018) (Figure 2), resulting in an overall postintervention slope of −0.725% per biweekly time point. Before implementation, the ICS rate changed from 9.41% to 9.30%, whereas after implementation it decreased from 12.10% to 9.27%. The estimated relative contribution of the intervention on the ICS rate was −22.69%, suggesting a potential beneficial association with the quality improvement program.

**TABLE 4:** Interrupted time series of intrapartum cesarean section.

	*β*	SE (*β*)	*t* value	*p* value
Intrapartum cesarean section rate				
Intercept *β*_0_	6.255	2.031	3.079	0.007
Baseline outcome trend *β*_1_	2.057	0.613	3.358	0.015
Level change after intervention *β*_2_	10.170	5.375	1.892	0.077
Trend change after intervention *β*_3_	−2.782	0.866	−3.212	0.018
Intrapartum cesarean section number				
Intercept *β*_0_	6.933	0.683	10.157	< 0.001
Baseline outcome trend *β*_1_	−0.279	0.110	−2.534	0.022
Level change after intervention *β*_2_	6.455	1.864	3.463	0.003
Trend change after intervention *β*_3_	−0.333	0.156	−2.142	0.048

*Note:* *β*_0_ is the baseline. *β*_1_ estimates the preintervention slope. *β*_2_ estimates the difference between the observed level just after the intervention started and that predicted by the preintervention slope. *β*_3_ estimates the difference in slopes between the preintervention (control) and intervention period.

### 3.4. Maternal Childbirth Experience

All 422 enrolled women completed the CEQ 2.0 with no missing items. After the implementation of the evidence‐based intrapartum care quality improvement program, the total score of childbirth experience in the intervention group (72.23 ± 9.76) was significantly higher than that in the control group (69.27 ± 8.84) (*p* < 0.05). In addition, the dimensional scores of own capacity, professional support, and participation in the intervention group were also significantly higher compared with those in the control group (*p* < 0.05) (Table 5).

**TABLE 5:** Maternal childbirth experience between the two groups **(** $\overset{¯}{x}\pm s$, score).

Dimension	Control group (*n* = 211)	Intervention group (*n* = 211)	*t* value	*p* value
Own capacity	20.23 ± 3.94	21.64 ± 3.42	−3.926	< 0.001
Professional support	16.87 ± 2.63	17.54 ± 3.31	−2.302	0.022
Perceived safety	15.32 ± 1.35	15.43 ± 1.43	−0.813	0.417
Participation	12.35 ± 2.27	14.65 ± 3.86	−7.461	< 0.001
Total score	69.27 ± 8.84	72.23 ± 9.76	−3.265	0.001

## 4. Discussion

This study represents one of the first applications of an ITS design to evaluate an evidence‐based intrapartum care quality improvement program in China. Our findings showed that the implementation of the program was associated with reductions in ICS rates and oxytocin use, shorter labor duration, and improved childbirth experience, indicating the potential value of integrating this midwife‐led intervention into routine perinatal care protocols. The intervention was designed to create a supportive birthing environment [3], respecting and responding to the individual preferences, needs, and values of the woman, ensuring that she is well informed and that her choices are honored [3, 5]. The intervention integrated continuity of professional midwifery care with family support, which may help reduce unnecessary medical interventions and facilitate physiological labor progression. Continuous midwife presence and individualized support may also enhance women’s confidence and participation during childbirth. These mechanisms are consistent with global recommendations for respectful and woman‐centered intrapartum care [3].

The observed reduction in ICS rates is consistent with evidence supporting midwifery continuity models. Key components of the program, including continuous one‐to‐one midwifery continuity support, individualized care plans, and family‐centered approaches, may contribute to improved clinical decision‐making by addressing nonprogressive labor through evidence‐based strategies rather than defaulting to medical intervention. This analysis is reinforced by the ITS design [25], which revealed a reversal from a preintervention ICS rate increase of 2.057% per biweekly time point to a postintervention declining trend. The negative contribution ratio confirms the intervention’s protective effect against unnecessary cesarean deliveries, corroborating findings from similar initiatives in diverse healthcare contexts [26]. In our study, the reduction in the rate of ICS deliveries observed postintervention suggests that the midwife‐led quality improvement program may have a significant impact on clinical decision‐making during labor and childbirth process. Continuous companionship and supportive childbirth services are recognized as beneficial for promoting the health outcomes of pregnant women and newborns [27]. The designated midwife in our study played an important role in implementing evidence‐based intrapartum care practices, as she directly influenced the provision of supportive labor care, responsive information feedback, and dynamic monitoring of labor progress. These measures could further enhance women’s sense of self‐control during labor and help promote normal childbirth for laboring women [28]. The upward trend in ICS rates observed prior to implementation may have been influenced by contextual factors, including healthcare system pressures during the COVID‐19 period in Shanghai. Increased workload, infection‐control policies, and staffing constraints may have influenced clinical decision‐making [29–31]. However, as these factors were not directly measured, this interpretation should be considered hypothesis generating rather than confirmatory. Future studies incorporating policy timelines, delivery volume, and staffing data would help clarify these contextual influences.

Meanwhile, our study also found lower oxytocin use among women receiving the intervention, which was consistent with the findings of Zahra et al. [32]. This observed reduction reflects the program’s effectiveness in promoting physiological labor progression. By integrating mobility encouragement, nonpharmacological pain management, and personalized pushing techniques, the intervention potentially mitigated anxiety‐induced dystocia, reduced the rate of oxytocin use during labor, and facilitated spontaneous vaginal delivery [33, 34]. In this study, we found that women in the intervention group experienced a shorter length of labor duration compared with the control group, suggesting that the implementation of this quality improvement program may contribute to promoting labor progress. This phenomenon can be attributed to the continuous labor support and woman‐centered childbirth care provided by the designated midwife [35, 36]. During the first stage of labor, women in the intervention group were allowed to move freely, choose various comfortable positions, and use birthing balls, breathing techniques, and diverse pain management methods. These practices helped alleviate labor pain and reduce adverse psychological reactions such as fear and anxiety during childbirth [37, 38]. In the second stage of labor, women were instructed to use appropriate pushing and breathing techniques. These strategies may help prevent excessive pushing and fatigue during labor, thereby potentially shortening the overall duration of labor [39, 40]. In the third stage of labor, the provision of midwife‐led early essential newborn care services, including delayed cord clamping, skin‐to‐skin contact, and early initiation of breastfeeding could facilitate placental detachment and expulsion, thus shortening the duration of labor stages [41].

Childbirth experience is a subjective physiological and psychological perception during labor and serves as a crucial indicator of the quality of intrapartum care [42]. In our study, the implementation of this evidence‐based program resulted in a significant improvement in overall childbirth experience scores, alongside enhancements in the dimensions of own capacity, professional support, and participation. Women in the intervention group received continuous emotional and physical care, as well as companion support from designated midwives. This supportive environment empowered women to take an active role during labor and childbirth, using techniques such as spontaneous or delayed pushing, which may help alleviate fatigue associated with childbirth [43]. Beyond that, midwives involved in the quality improvement program performed timely and ongoing assessments of women’s personal needs and psychological states, providing interventions such as delayed cord clamping and early skin‐to‐skin contact between mothers and their newborns. These measures enhance women’s autonomy during childbirth, facilitate informed decision‐making, and promote adaptation to their new maternal roles, thus fostering a positive childbirth experience [44]. Mojgan et al. [45] also found that timely assessment and addressing the childbirth needs of women can significantly improve their birth experiences. As such, midwives should engage in dynamic assessments in clinical practice to identify women’s health needs and deliver individualized continuous labor care. The integration of companion support, emotional support, and continuity of midwifery‐led care significantly enhances the quality of intrapartum care, contributing to fewer medical interventions and greater maternal satisfaction [3, 27].

### 4.1. Implications for Nursing Management

Findings from this study carry direct implications for nursing management in intrapartum care settings. Nurse managers should prioritize establishing midwife‐led continuity of care models with adequate staffing to sustain one‐to‐one labor support throughout the entire childbirth process. Institutionalizing structured, competency‐based training programs focused on standardized labor monitoring, restrictive oxytocin use, and individualized pushing guidance is essential for delivering high‐quality intrapartum care consistently across shifts. Beyond midwifery staffing and training, fostering an organizational culture that encourages woman‐centered labor management, respectful communication, and women’s active participation in decision‐making remains a key responsibility. Dedicated midwifery care resources, including single labor and delivery rooms and nonpharmacological pain relief equipment, should be integrated into resource planning to support evidence‐based intrapartum practice at the bedside. Routine audit and feedback mechanisms should also be embedded into nursing management systems to monitor midwifery care fidelity and sustain quality improvement outcomes over time.

### 4.2. Strengths and Limitations

A major strength of this study is the use of an ITS design, which allows the evaluation of intervention effects while accounting for pre‐existing temporal trends. Segmented regression analysis enabled the assessment of both level and slope changes following implementation. However, several limitations should be considered. First, as a single‐center quasiexperimental study without an external control site, residual confounding from concurrent practice changes cannot be ruled out. Second, selection bias within time intervals may have occurred because patient characteristics may have varied across biweekly periods. Third, documentation practices may have changed over time following implementation of the quality improvement program, potentially influencing measured outcomes. Fourth, the intensive training and monitoring associated with the intervention may have introduced a Hawthorne effect, whereby staff modified behavior due to awareness of being observed. Fifth, the use of aggregated biweekly data may obscure short‐term fluctuations. Finally, the single tertiary hospital setting may limit generalizability to other maternity care contexts. Multicenter studies with external comparison sites are needed to confirm these findings.

## 5. Conclusion

This study developed and implemented an evidence‐based intrapartum care quality improvement program that was associated with reduced ICS rates, decreased oxytocin use, shorter labor duration, and improved childbirth experience. These findings support the potential value of midwife‐led continuity care models in promoting physiological childbirth and improving women’s experiences. Given the quasiexperimental design, further multicenter studies with controlled comparisons are warranted to confirm effectiveness and assess scalability. Future research should focus on multisite implementation, contextual adaptation, and long‐term evaluation to strengthen the evidence base for wider adoption of this intrapartum care quality improvement program.

## Author Contributions

Wenli Zhu and Chunyi Gu conceptualized the study and proposed the methodology. Wenli Zhu, Yaming Dai, Hui Min, Chunxiang Zhu, and Xiaojiao Wang collected data and performed formal analysis. Wenli Zhu, Chunyi Gu, and Yaming Dai accessed and verified the data. Wenli Zhu and Hui Min wrote the original draft. Chunyi Gu reviewed and edited the manuscript.

## Funding

The study was funded by the National Natural Science Foundation of China (Grant no. 72004029) and the Shanghai Municipal Health Commission (Grant no. 202040097).

## Disclosure

The funder had no role in the study design, data analysis, interpretation of results, writing of the report, and in the decision to submit the article for publication. All authors had full access to all data in the study, and the corresponding author had final responsibility for the decision to submit for publication.

## Ethics Statement

This study was approved by the Ethics Committee of the Obstetrics and Gynecology Hospital of Fudan University (OGHEC2021107). Participants gave informed consent to participate in the study before taking part.

## Conflicts of Interest

The authors declare no conflicts of interest.

## Data Availability

The data that support the findings of this study are available from the corresponding author upon reasonable request.
